# Pengzhenrongella phosphoraccumulans sp. nov., isolated from high Arctic glacial till, and emended description of the genus Pengzhenrongella

**DOI:** 10.1099/ijsem.0.006368

**Published:** 2024-05-09

**Authors:** Jialin Xie, Lvzhi Ren, Ziyan Wei, Xiaoya Peng, Kun Qin, Fang Peng

**Affiliations:** 1 China Center for Type Culture Collection (CCTCC), College of Life Sciences, Wuhan University, Wuhan 430072, PR China; 2 Key Laboratory of Polar Environment Monitoring and Public Governance (Wuhan University), Ministry of Education, Wuhan, PR China

**Keywords:** high-Arctic glacial till, *Pengzhenrongella phosphoraccumulans*, Svalbard Archipelago

## Abstract

A yellow pigmented, Gram-stain-positive, motile, facultatively anaerobic and irregular rod-shaped bacteria (strain M0-14^T^) was isolated from a till sample collected from the foreland of a high Arctic glacier near the settlement of Ny-Ålesund in the Svalbard Archipelago, Norway. Phylogenetic analysis based on 16S rRNA gene sequence comparisons revealed that M0-14^T^ formed a lineage within the family *Cellulomonadaceae*, suborder *Micrococcineae*. M0-14^T^ represented a novel member of the genus *Pengzhenrongella* and had highest 16S rRNA gene sequence similarity to *Pengzhenrongella sicca* LRZ-2^T^ (97.3 %). Growth occurred at 4–25 °C (optimum 4–18 °C), at pH 6.0–9.0 (optimum pH 7.0), and in the presence of 0–5 % (w/v) NaCl. The predominant menaquinone was MK-9(H_4_) and the major fatty acids were anteiso-C_15 : 0_, C_16 : 0_ and summed feature 3 (comprising C_16 : 1_ω7*c* and/or C_16 : 1_ω6*c*). The major polar lipids were phosphatidylglycerol, phosphatidylinositol mannosides, phosphatidylinositol, one undefined phospholipid and five undefined phosphoglycolipids. The cell-wall diamino acid was l-ornithine whereas rhamnose and mannose were the cell-wall sugars. Polyphosphate particles were found inside the cells of M0-14^T^. Polyphosphate kinase and polyphosphate-dependent glucokinase genes were detected during genomic sequencing of M0-14. In addition, the complete *pstSCAB* gene cluster and *phnCDE* synthesis genes, which are important for the uptake and transport of phosphorus in cells, were annotated in the genomic data. According to the genomic data, M0-14^T^ has a metabolic pathway related to phosphorus accumulation. The DNA G+C content of the genomic DNA was 70.8 %. On the basis of its phylogenetic relationship, phenotypic properties and chemotaxonomic distinctiveness, strain M0-14^T^ represents a novel species of the genus *Pengzhenrongella*, for which the name *Pengzhenrongella phosphoraccumulans* sp. nov. is proposed. The type strain is M0-14^T^ (= CCTCC AB 2012967^T^ = NRRL B-59105^T^).

The suborder *Micrococcineae*, within the family *Cellulomonadaceae*, was first proposed by Stackebrandt *et al.* [1]. The genus *Pengzhenrongella* was proposed by Kim *et al.* [2]. Members of the genus *Pengzhenrongella* have been isolated from tundra soil and glacial till. Members of the genus *Pengzhenrongella* are characterised as facultatively anaerobic, non-sporulating and short rod-shaped, with high DNA G+C contents (over 70 %) and MK-9(H_4_) as the predominant menaquinone. According to Kim, the most closely related genera to the genus *Pengzhenrongella* within the suborder *Micrococcineae* include *Luteimicrobium*, *Cellulomonas*, *Oerskovia*, *Actinotalea*, *Sediminihabitans* and *Sanguibacter*. In this study, we aimed to characterise and determine the taxonomic position of a novel strain, M0-14^T^, isolated from high Arctic glacial till sample. The GenBank/EMBL/DDBJ accession numbers of 16S rRNA gene sequence and genome sequences of M0-14^T^ (= CCTCC AB 2012967^T^ = NRRL B-59105^T^) are PP134910 and CP144210, respectively.

M0-14^T^ was isolated from a till sample collected from the foreland of a high Arctic glacier Midtre Lovénbreen (78° 53.704′ N, 12° 05.262′ E) near the settlement Ny-Ålesund in the Svalbard Archipelago, Norway, in July 2012. The sample was subjected to standard spread plate culture using Reasoner’s 2A medium (R2A; BD). The plates were incubated at 4 °C for 4–6 weeks. Single colonies on these plates were purified by transferring them onto new plates. Strain M0-14^T^ was preserved by lyophilisation.

The 16S rRNA gene sequence of M0-14^T^ was amplified by PCR with primers 27F and 1492R [3]. The purified PCR products were sequenced by GenScript (Nanjing, PR China). After sequencing, an incomplete sequence of the 16S rRNA gene (1389 bp) was assembled and obtained. To determine an approximate phylogenetic affiliation of the novel strain, GenBank and Ezbiocloud servers [4] were used to acquire the sequences of related taxa. Multiple alignments were performed and by using the clustal_x programme [5]. Phylogenetic trees based on 16S rRNA gene comparison were reconstructed by using the neighbour-joining [6], maximum-likelihood [7] and maximum-parsimony methods [8] with the software package mega11 [9] with bootstrap values based on 1000 replications and are shown in Figs 1, S1 and S2 (available in the online version of this article).

**Fig. 1.**
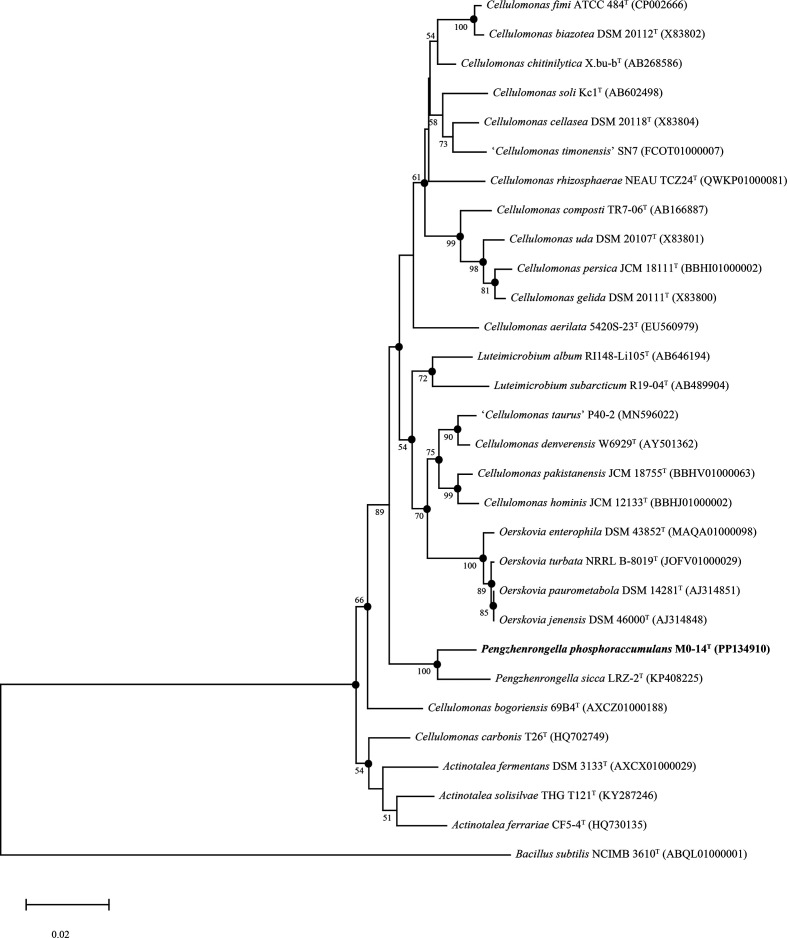
Neighbour-joining phylogenetic tree reconstructed from a comparative analysis of 16S rRNA gene sequence showing the relationships of strain M0-14^T^ with related taxa. Bootstrap values (expressed as percentage of 1000 replications) above 50 are shown at the branch points. The dots indicate that the corresponding branches were recovered in the trees reconstructed with the maximum-likelihood tree and maximum-parsimony algorithms. The sequence of *Bacillus subtilis* NCIMB 3610^T^ was used as an outgroup. Bar, 0.02 substitutions per nucleotide position.

The genomic DNA of M0-14^T^ was extracted and purified using a bacterial genomic DNA Mini kit (Qiagen) for whole-genome sequencing. The genome of M0-14^T^ was sequenced by Wuhan Nextomics Corporation (Wuhan, Hubei Province) using the PACBIO RS II platform [10]. High molecular weight genomic DNA was extracted from given samples. Agarose gel electrophoresis NanoDrop and Qubit were used to guarantee the purity, quantity and size of DNA. The genomic DNA was randomly interrupted using an ultrasonicator (S220 Covaris). Then the large fragments of DNA were enriched and purified using magnetic beads. A stem–loop sequencing link was ligated at both ends of the DNA fragments and exonucleases were used to remove fragments that failed to join. A Bioanalyzer High Sensitivity Kit (model 2100 Agilent) was used to ensure good-quality paired reads. All good-quality paired reads were assembled by using the HGAP2.2.3 analysis process [11].

After assembly, the genome sequencing of M0-14^T^ yielded a genome of 4 958 599 bp with a DNA G+C content of 70.8 %. The DNA G+C content is similar to that of the other member of the genus*, Pengzhenrong sicca* LRZ-2^T^, (72.4 %) and similar to those of the members of other genera of the same family. The genome of M0-14^T^ contained one contig and had a total of 4827 predicted genes, of which there were 9 rRNA genes and 46 tRNA genes. There were three copies of the 1516 bp length 16S rRNA in the genome. The similarity of the sequences of the 16S rRNA genes from the genome and that of the PCR-determined sequences was 100 %, 99.9 and 99.9  %. The genome of M0-14^T^ was analysed as described by Chun *et al.* [12]. The average nucleotide identity (ANI) and digital DNA–DNA hybridisation (dDDH) scores were calculated using ANI Calculator (www.ezbiocloud.net/tools) and the Genome-to-Genome Distance Calculator (GGDC 3.0; https://ggdc.dsmz.de/ggdc.php#) [13,14], respectively. As shown in Table S2, the ANI and dDDH values between the genomes of M0-14^T^ and closely related strains were 80.0–73.8 % and 23.1–19.3 %, respectively, which are lower than the species thresholds of 95–96 % [15] and 70 % [16]. These data indicated that M0-14^T^ represented a different species from *Pengzhenrongella sicca* LRZ-2^T^ and those closely related strains of members of the genera *Cellulomonas*, *Actinotalea* and *Luteimicrobium*. A whole-genome-based phylogenetic tree was reconstructed using CVTree 3.0 with a K value of 12 [17]. In the whole-genome-based composition of vectors (CV) phylogenomic tree (Fig. S3), M0-14^T^ formed a coherent cluster with *P. sicca* LRZ-2^T^. Compared with members of the same genus or other genera in the same family, the most striking difference is that the genome of M0-14^T^ contained genes for the synthesis and transport pathway of polyphosphate particles. According to the comparison results from Swiss-prot database, M0-14^T^ had a polyphosphate kinase gene and two polyphosphate-dependent glucokinase genes, which are responsible for the synthesis of polyphosphate particles. On the basis of the genomic annotation, it is speculated that the uptake and transport of phosphorus in M0-14^T^ are mainly through the *pstSCAB* gene cluster and/or *phnCDE* transport systems. Through the study of the whole genome information of M0-14^T^, it was found that its functional proteins are mostly related to material metabolism, signal transduction, replication, transcription, translation and repair of genetic material, which will help it adapt to the extreme polar environment.

Cell morphology was examined by phase-contrast (BX51; Olympus) and transmission electron microscopy (H-8100; Hitachi). The Gram staining of cells was carried out according to the classical Gram procedure described by Doetsch [18]. Growth of M0-14^T^ was evaluated at 18 °C on R2A agar (BD), tryptic soy agar (TSA, BD), nutrient agar (NA, BD), MacConkey agar (BD) and glucose alkaline medium (GAM) which consisted of two parts. GAM solution A contained the following, dissolved in 800 ml distilled water and sterilised: glucose (10 g), peptone (Difco; 5 g), yeast extract (Difco ; 5 g), K_2_HPO_4_ (1 g) and MgSO_4_·7H_2_O (0.2 g). GAM solution B contained 40 g NaCl and 10 g Na_2_CO_3_ dissolved in 200 ml distilled water and sterilised. The two solutions were then mixed. Solid medium was prepared by adding agar (2 %, w/v) to GAM solution A before sterilisation [19]. Growth at various temperatures (4, 10, 18, 22, 25, 28, 37 and 42 °C) was measured on R2A agar and the optimum pH for growth was examined in R2A broth adjusted to various pH values (pH 4.0–11.0, at intervals of 1.0 pH unit). Tolerance of NaCl was tested in R2A broth with different NaCl concentrations (0–9 %, w/v, at intervals of 1 % unit). Gliding motility was investigated as described by Bowman [20]. Growth under anaerobic conditions was determined after incubating the strain in a GasPak (Oxoid) jar at 18 °C for a month on R2A agar supplemented with 0.1 % (w/v) potassium nitrate.

Tests for degradation of cellulose, starch, xylan, DNA, casein, tyrosine, xanthine and hypoxanthine were performed and evaluated after 7 days. Hydrolysis of chitin was tested as described by Rodriguez-Kabana *et al.* [21]. Catalase activity was determined by assessing bubble production in 3 % (v/v) H_2_O_2_ and oxidase activity was determined using 1 % (w/v) tetramethyl-*p*-phenylenediamine [22]. Susceptibility to antibiotics was tested on R2A plates by using the disc diffusion method and discs containing the following: ampicillin (10 µg), chloramphenicol (30 µg), streptomycin (10 µg), tetracycline (30 µg), polymyxin B (300U IE^−1^), rifampicin (5 µg), vancomycin (30 µg), gentamicin (10 µg), nalidixic acid (30 µg), trimethoprim (300 µg), penicillin G (10 U IE^−1^), kanamycin (30 µg), amikacin (30 µg), compound sulfamethoxazole (23.75 µg), erythromycin (15 µg), novobiocin (30 µg), nitrofurantoin (300 µg), sulphamethoxazole (50 µg), sulfafurazole (100 µg), ofloxacin (5 µg), and clindamycin (2 µg). In addition, biochemical phenotypic tests were carried out using API 20NE, API 20E, API 50CH, API ZYM test kits according to the instructions of the manufacturer (bioMérieux).

Purified cell-wall preparations were obtained as described by Schleifer and Kandler [23]. Amino acids and peptides in cell-wall hydrolysates were analysed by one-dimensional TLC (silica gel plates, Merck) using methanol/pyridine/water/10 M HCl (32 : 4 : 7 : 1, v/v/v/v) at 25 °C. Cell-wall sugars were analysed as described by Staneck and Roberts [24]. Respiratory quinones were extracted and identified by HPLC (UltiMate 3000, Dionex) as described by Xie and Yokota [25]. For cellular fatty acids analysis, M0-14^T^ and reference strains, *P. sicca* LRZ-2^T^, *Cellulomonas cellasea* KACC 20548^T^ and *Cellulomonas chitinilytica* DSM 17922^T^ were cultured on R2A medium at 22 °C, whereas *Actinotalea fermentans* KACC 20763^T^ and *Cellulomonas bogoriensis* KACC 20567^T^ were cultured on BHI agar (BD) at 22 °C. The cellular fatty acids were saponified, methylated, extracted and identified by GC (6890 N, Agilent) according to the standard protocol of the Sherlock Microbial Identification System (MIDI Sherlock version 6.0, MIDI database TSBA6) [26]. Polar lipids were extracted using the procedures described by Minnikin *et al.* [27] and identified using two-dimensional TLC as described by Tindall [28].

The cells of M0-14^T^ were Gram-stain-positive, facultatively anaerobic, motile and irregular rod shaped (0.2–0.3×0.3–1.1 µm). Cells were reproduced by budding or cell division and had a lateral flagellum visible on TEM examination (Fig. 2). Polyphosphate particles were found in the ultrathin section of cells of M0-14^T^ under TEM and determined using an energy dispersive spectrometer (Figs 2 and S4). Colonies on R2A agar were circular, convex, entire, smooth and yellow in colour within 3–5 days at 18 °C. Growth occurred on R2A, TSA, NA, MacConkey agar but not on GAM. The temperature range for growth was 4–25 °C, with an optimum at around 4–18 °C. Growth occurred at pH values between 6.0 and 9.0, with an optimum at pH 7.0. Growth occurred well without NaCl supplementation but also in the presence of 1–5 % (w/v) NaCl. The strain showed a positive result for catalase activity and a negative one for oxidase activity. The bacterium could hydrolyse starch and casein but not DNA, cellulose, chitin, xylan, tyrosine, xanthine or hypoxanthine. The phenotypic characteristics that differentiate M0-14^T^ from related species are listed in Table 1. M0-14^T^ could not hydrolyse cellulose, but the most closely related strain, LRZ-2^T^, and reference strains of members of the genus *Cellulomonas* could. M0-14^T^ could utilise glycerol and d-ribose. These data indicated that the phenotypic characteristics of M0-14^T^ were distinct from those of its closely related species.

**Fig. 2.**
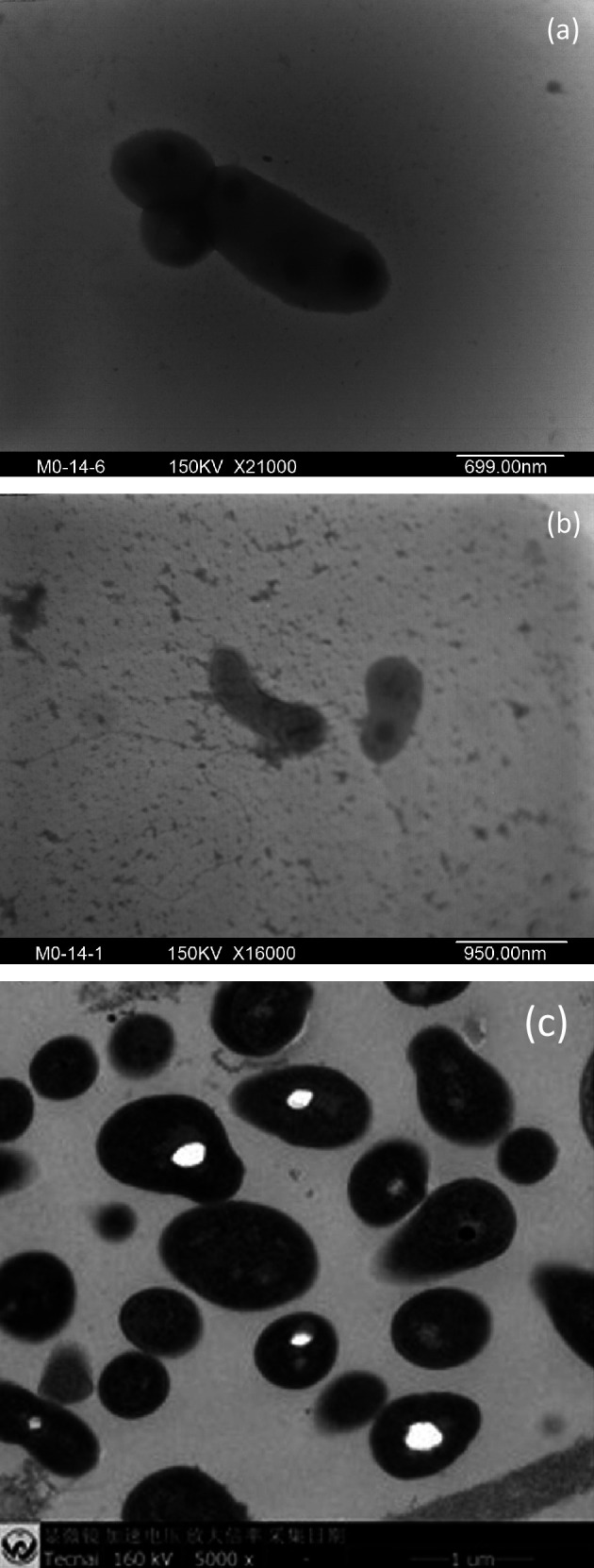
Transmission electron microscopy of M0-14^T^ cells showing bud formation (**a**) and lateral flagellum (**b**). An ultrathin section of M0-14^T^ cells under TEM showing polyphosphate particles (**c**). Bars, 699 nm (**a**), 950 nm (**b**) and 1 µm (**c**).

**Table 1.** Differential phenotypic characteristics of M0-14^T^ and type strains of species of the genera *Pengzhenrongella*, *Cellulomonas* and *Actinotalea*. Data were obtained during this study unless indicated Strains: 1, M0-14^T^; 2, *P. sicca* LRZ-2^T^; 3, *C. cellasea* KACC 20548^T^; 4, *A. fermentans* KACC 20763^T^; 5, *C. chitinilytica* DSM 17922^T^; 6, *C. bogoriensis* KACC 20567^T^. +, Positive; −, negative; w, weakly positive; nd, no data available.

Characteristics	1	2	3	4	5	6
Motility	+	−	−	−	−	+
Morphology	Rod	Rod	Rod	Rod	Rod	Rod
Nitrate reduction	−	nd	−	−	+	−
Voges–Proskauer tests	+	+	+	+	+	−
Catalase	+	+	−	−	+	+
Hydrolysis of:						
Cellulose	−	+	+	−	+	+
Starch	+	+	+	+	+	−
Assimilation of:						
d-Glucose	w	+	−	−	+	−
l-Arabinose	w	+	−	−	+	−
d-Mannose	−	−	−	−	+	−
d-Mannitol	−	−	−	−	+	−
*N*-Acetyl-glucosamine	−	−	−	−	+	−
d-Maltose	−	−	−	−	+	−
Potassium gluconate	−	−	−	−	w	−
Acid production from:						
Glycerol	+	−	−	−	w	−
l-Arabinose	+	+	+	+	+	−
d-Ribose	+	−	−	−	−	−
d-Galactose	+	−	+	−	+	−
l-Rhamnose	−	−	+	−	+	−
d-Mannitol	+	+	+	−	+	−
d-Sorbitol	−	−	−	−	+	−
d-Cellobiose	+	+	+	−	+	+
d-Maltose	+	+	+	−	+	+
d-Sucrose	+	+	+	−	+	+
d-Trehalose	w	−	+	−	+	+
Starch	+	−	+	−	+	−
Glycogen	+	−	+	−	+	−
Gentiobiose	+	−	+	−	+	+
d-Arabitol	+	−	−	−	+	−
API ZYM test results						
Esterase (C4)	−	−	w	nd	−	w
Esterase lipase (C8)	−	−	w	nd	−	w
Acid phosphatase	w	+	−	nd	−	−
Naphthol-AS-BI-phosphohydrolase	w	+	+	nd	+	+
α-Galactosidase	w	−	−	nd	−	−
β-Galactosidase	w	+	−	nd	w	−
β-Glucosidase	+	−	+	nd	−	−
*N*-Acetyl-β-glucosaminidase	−	−	w	nd	w	−
DNA G+C content (%)	70.8	72.4	75*	76†	73.6‡	71.5§

§: *Data from Stackebrandt *et al* [29].,【】1979; : Data from Yi , 2007; : Data from Yoon , 2008; : Data from Jones , 2005.

†Data from Yi *et al.* 2007 [30].

‡Data from Yoon *et al.* 2008 [31].

§Data from Jones *et al.* 2005 [32].

The peptidoglycan composition of M0-14^T^ corresponded to type A4β: it contained l-ornithine–d-glutamic acid (l-Orn–d-Glu), which is the composition reported for the most closely related species, *P. sicca* LRZ-2^T^. The cell-wall sugars of M0-14^T^ were rhamnose, mannose, xylose, ribose, glucose and 6-deoxytalose, which are consistent with those of *P. sicca* LRZ-2^T^ and members of the genus *Cellulomonas.* The cellular fatty acid profiles of M0-14^T^ and its reference strains are shown in Table S1. The major fatty acids of M0-14^T^ were anteiso-C_15 : 0_ (36.9 %), C_16 : 0_ (20.2 %) and summed feature 3 (comprising C_16 : 1_ω7*c* and/or C_16 : 1_ω6*c*, 10.9 %). The fatty acid profile was similar to that of *P. sicca* LRZ-2^T^ and members of the genus *Cellulomonas,* but there were differences in the proportions of some fatty acids, especially the high content of summed feature 3 of M0-14^T^. The isoprenoid quinone in M0-14^T^ was a tetrahydrogenated menaquinone with nine isoprene units [MK-9(H_4_)], which is consistent with the respiratory quinone found in *P. sicca* LRZ-2^T^ and members of the genus *Cellulomonas*. The polar lipids detected were phosphatidylglycerol (PG), phosphatidylinositol mannosides (PIM) and phosphatidylinositol (PI). Five unknown phosphoglycolipids (PGL1–PGL5), three unknown phospholipids (PL1–PL3), one unknown aminophospholipid (APL), one unknown aminolipid (AL) and two unknown lipids (L1–L2) were also detected (Fig. S5). The composition of polar lipids was similar to those of members of the genus *Cellulomonas*. Compared with LRZ-2^T^, both strains contained PI and PIM, but M0-14 did not contain DPG and had a low PG content and a high PL content.

In conclusion, On the basis of the 16S rRNA gene sequence and phylogenetic relationship, physiological characteristics and chemotaxonomic properties of strain M0-14^T^ presented in this study, it represents a novel species of the genus *Pengzhenrongella*, for which the name *Pengzhenrongella phosphoraccumulans* sp. nov. is proposed.

## Emended description of *Pengzhenrongella* Kim *et al*. 2021

The description is based on that given by Kim *et al*. [2]. The major fatty acids are anteiso-C_15 : 0_, iso-C_16 : 0_, anteiso-C_15 : 1_A and anteiso-C_17 : 0_. C_16 : 0_ and summed feature 3 (comprising C_16 : 1_*ω*7*c* and/or C_16 : 1_ω6*c*) may contribute significantly to the profile.

## Description of *Pengzhenrongella phosphoraccumulans* sp. nov.

*Pengzhenrongella phosphoraccumulans* (phos.phor.ac.cu’mu.lans. L. masc. n. *phosphorus*, phosphorus (P); L. pres. part. *accumulans*, accumulating; N.L. part. adj. *phosphoraccumulans*, phosphorus-accumulating).

Cells are Gram-stain-positive, facultatively anaerobic, motile and irregular rod shaped (0.2–0.3×0.3–1.1 µm). Cells reproduce by budding or cell division and have a lateral flagellum. Colonies on R2A agar are circular, convex, entire, smooth, translucent and yellow in colour within 3–5 days at 18 °C. Growth occurs on R2A, TSA, NA and MacConkey agar but not on GAM. The temperature range for growth is 4–25 °C, with an optimum at around 4–18 °C. No growth occurs at 37–42 °C. Grows well without NaCl supplementation but also in the presence of 1–5 % (w/v) NaCl and at pH 6.0–9.0 (optimum, pH 7.0). Catalase-positive and oxidase-negative. Can hydrolyse starch and casein but not DNA, cellulose, chitin, xylan, tyrosine, xanthine and hypoxanthine. According to the API 20E and API 20NE kits, positive for β-galactosidase and Voges–Proskauer tests and can hydrolyse aesculin but negative for nitrate reduction, indole production and urease. H_2_S is not produced from cysteine and does not hydrolyse gelatin. There is no arginine dihydrolase or tryptophane deaminase. Acid is produced from no substrates and glucose and arabinose are assimilated weakly. According to API 50CH, acid is produced from glycerol, l-arabinose, d-ribose, d-xylose, d-galactose, d-glucose, d-fructose, d-mannose, d-mannitol, arbutin, aesculin, salicin, d-cellobiose, d-maltose, d-sucrose, d-trehalose, starch, glycogen, gentiobiose, d-turanose and d-arabitol and acid is not produced from the remaining substrates in the test kit. For API ZYM tests, the results for leucine arylamidase, α-glucosidase and β-glucosidase are positive. Results for acid phosphatase, naphthol-AS-BI-phosphohydrolase, α-galactosidase and β-galactosidase are weakly positive. Results for alkaline phosphatase, esterase (C4), esterase lipase (C8), lipase (C14), valine arylamidase, cystine arylamidase, trypsin, α-chymotrypsin, *N*-acetyl-β-glucosaminidase, β-glucuronidase, α-mannosidase and α-fucosidase are negative. Sensitive to gentamicin, penicillin G, kanamycin, rifampicin, ampicillin, nitrofurantoin, vancomycin, tetracycline and streptomycin. Resistant to chloramphenicol, polymyxin B, nalidixic acid, trimethoprim, amikacin, compound sulfamethoxazole, erythromycin, novobiocin, sulphamethoxazole, sulfafurazole, ofloxacin and clindamycin. The peptidoglycan type is A4β: containing l-ornithine-d-glutamic acid, and l-ornithine is the cell-wall diamino acid, rhamnose, mannose, xylose, ribose, glucose and 6-deoxytalose are the cell-wall sugars. Anteiso-C_15 : 0_, C_16 : 0_ and summed feature 3 (comprising C_16 : 1_ω7*c* and/or C_16 : 1_ω6*c*) are the major fatty acids and MK-9(H_4_) is the predominant menaquinone. The major polar lipids are phosphatidylglycerol, phosphatidylinositol mannosides, phosphatidylinositol, one undefined phospholipid and five undefined phosphoglycolipids.

The type strain, M0-14^T^ (= CCTCC AB 2012967^T^ = NRRL B-59105^T^), was isolated from a till sample collected from the foreland of a high Arctic glacier Midtre Lovénbreen near the settlement of Ny-Ålesund in the Svalbard Archipelago, Norway. The DNA G+C content of the type strain is 70.8 %.The GenBank/EMBL/DDBJ accession numbers for the 16S rRNA gene and genome sequences of strain M0-14^T^ are PP134910 and CP144210, respectively.

## supplementary material

10.1099/ijsem.0.006368 Uncited Supplementary Material 1.

## Abbreviations

ANI: average nucleotide identity
dDDH: digital DNA–DNA hybridisation
