# Supplementary material for: Pengzhenrongella phosphoraccumulans sp. nov., isolated from high Arctic glacial till, and emended description of the genus Pengzhenrongella
Source: Int J Syst Evol Microbiol. 2024 May 9;74(5):006368. doi: 10.1099/ijsem.0.006368 (PMC11165906; doi:10.1099/ijsem.0.006368)
Supplement: Uncited Supplementary Material 1. [file ijsem-74-06368-s001.pdf]

Pengzhenrongella phosphoraccumulans sp. nov., isolated from high Arctic glacial till, and emended description of the genus *Pengzhenrongella*

Jialin Xie[1], Lvzhi Ren[1], Ziyan Wei[1], Xiaoya Peng[1], Kun Qin[1], Fang Peng[1,2,]*

[1]China Center for Type Culture Collection (CCTCC), College of Life Sciences, Wuhan University, Wuhan 430072, China

[2]Key Laboratory of Polar Environment Monitoring and Public Governance (Wuhan University), Ministry of Education

Running title: *Pengzhenrongella phosphoraccumulans* sp. nov., isolated from high Arctic glacial till, and emended description of the genus *Pengzhenrongella*

Subject category: New Taxa – *Actinomycetota*

*Corresponding Author: Fang Peng

E-mail: pf-cctcc@whu.edu.cn

Tel: +86-27-68752319

Fax: +86-27-68754833

Sequence deposited: The GenBank/EMBL/DDBJ accesstion number of 16S rRNA gene sequence of strain M0-14^T (= CCTCC AB 2012967^T = NRRL B-59105^T) is PP134910. The GenBank/EMBL/DDBJ accesstion number of genome sequence of strain M0-14^T is CP144210.

Fig. S1. Maximum-likelihood phylogenetic tree constructed from a comparative analysis of 16S rRNA gene sequence showing the relationships of strain M0-14^{T} with other related taxa. Bootstrap values (expressed as percentage of 1000 replications) of above 50 are shown at the branch point. The sequence of *Bacillus subtilis* NCIMB 3610^{T} was used as an outgroup. Bar, 0.02 substitutions per nucleotide position.

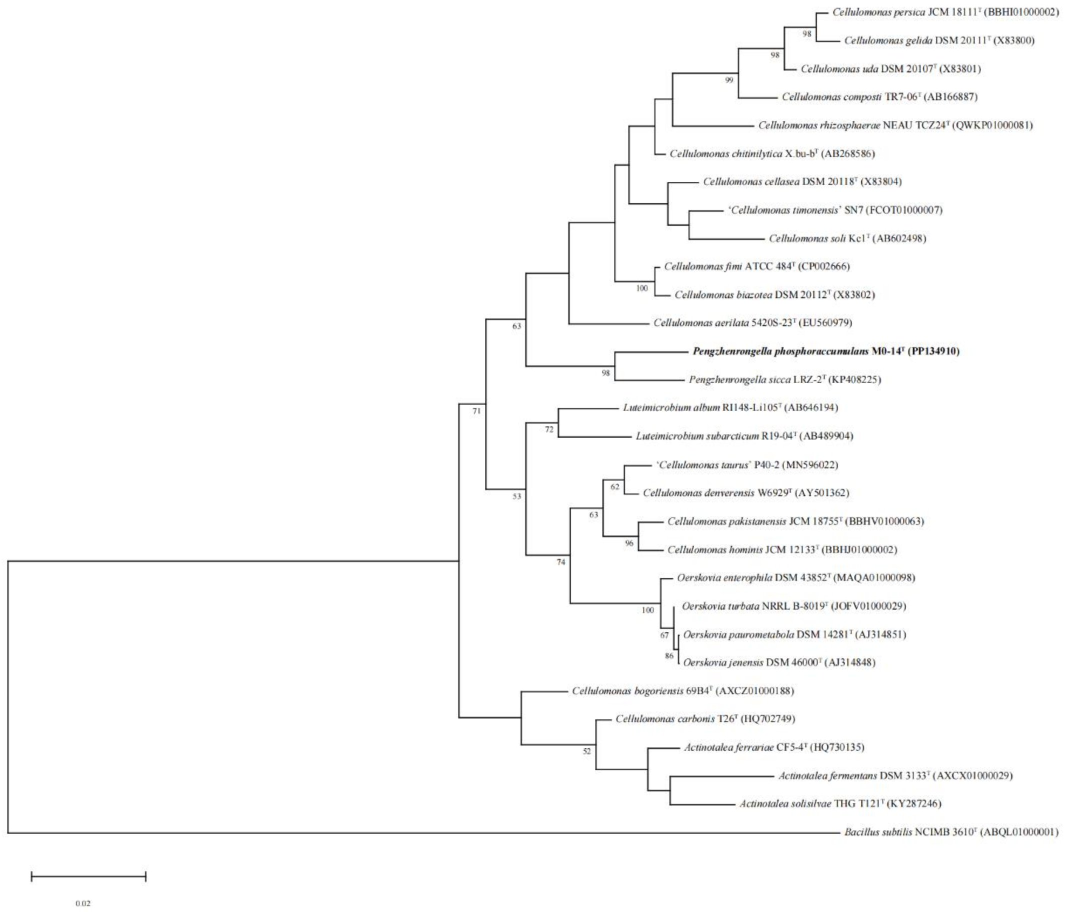

(Fig. S1)

Fig. S2. Maximum-parsimony phylogenetic tree constructed from a comparative analysis of 16S rRNA gene sequence showing the relationships of strain M0-14^T with other related taxa. Bootstrap values (expressed as percentage of 1000 replications) of above 50 are shown at the branch point. The sequence of *Bacillus subtilis* NCIMB 3610^T was used as an outgroup. Bar, 3.00 substitutions per nucleotide position.

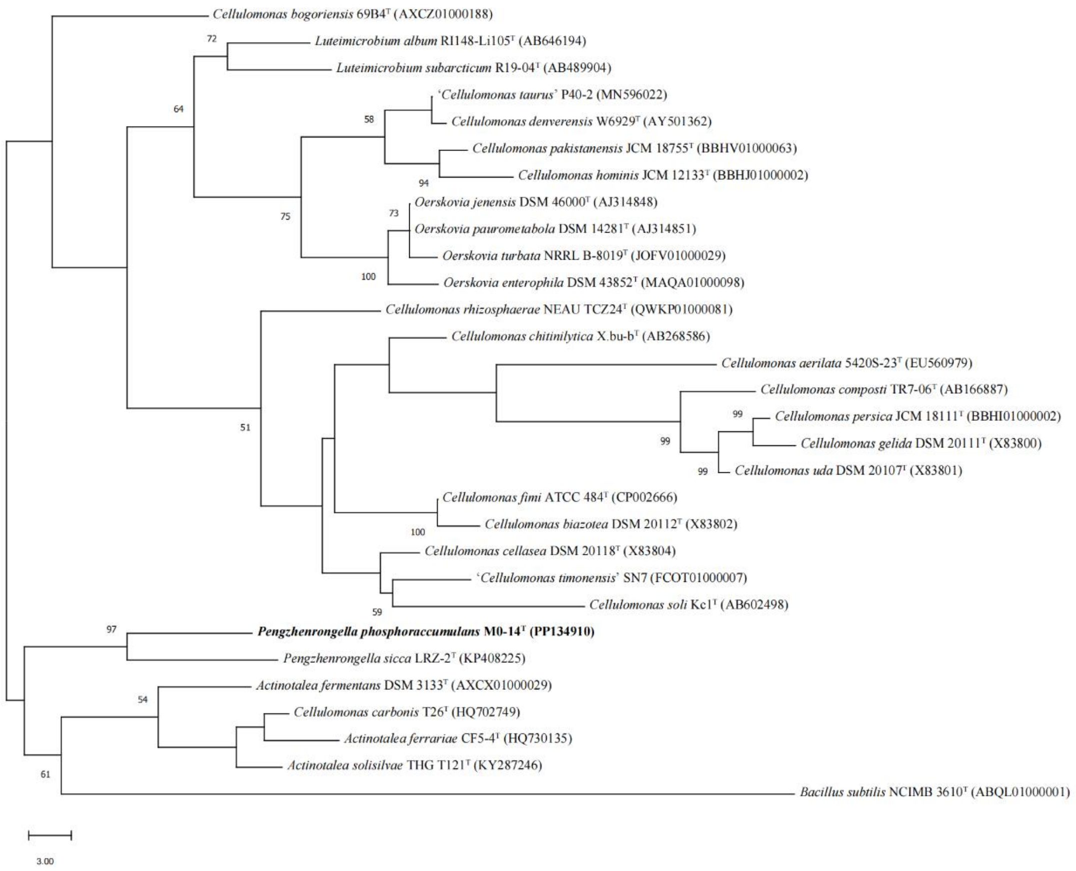

(Fig. S2)

Fig. S3. CV phylogenomic tree showing the relationships between strain M0-14^T and closely related 9 strains. K value, 12. The sequence of *Bacillus cereus* ATCC 10987 was used as an outgroup.

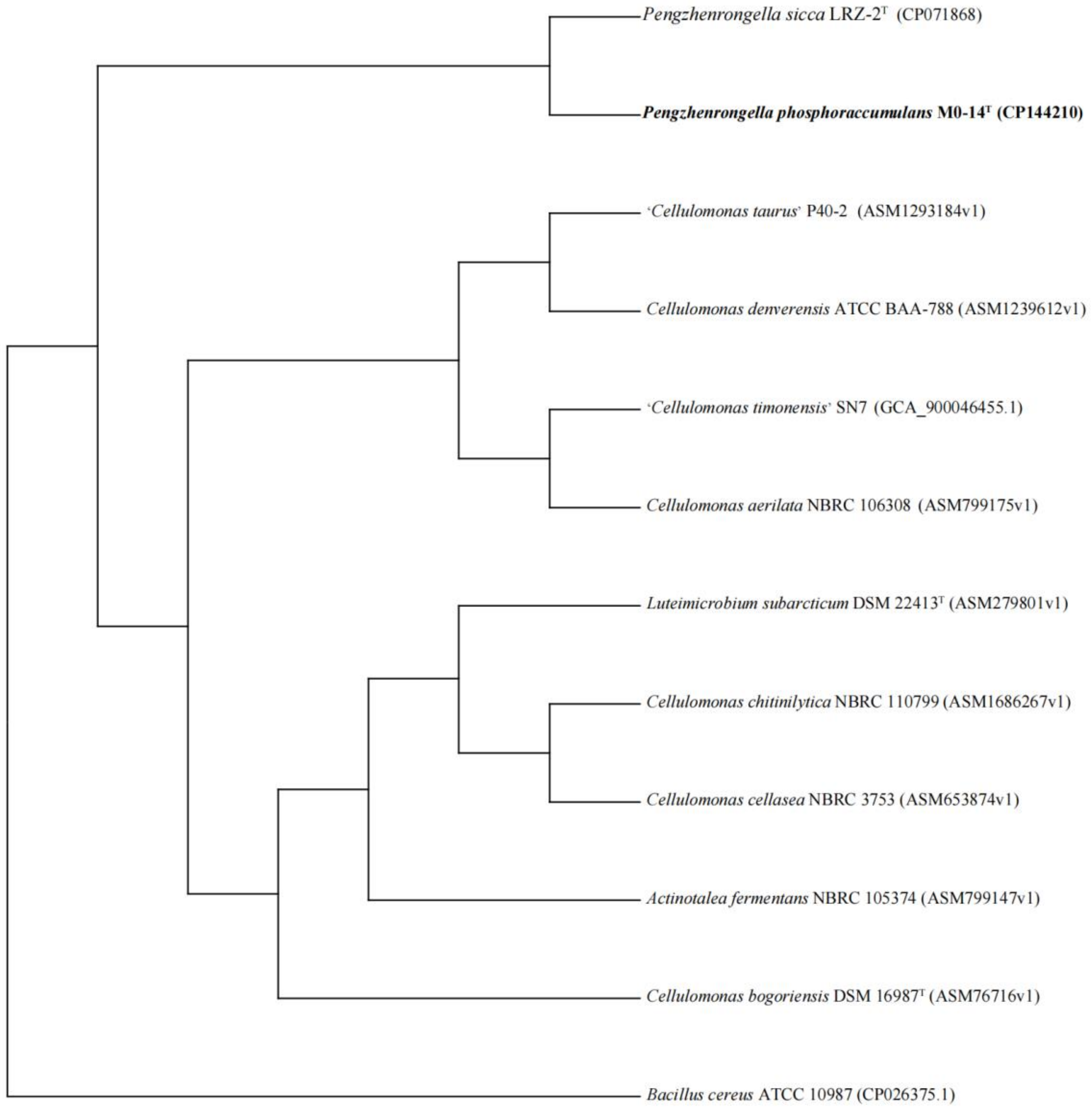

(Fig. S3)

Fig. S4. The detection of voids (A) caused by ultrathin sections and intracellular particles (B) of strain M0-14^{T} cells revealed that the particles were mainly polphosphate.

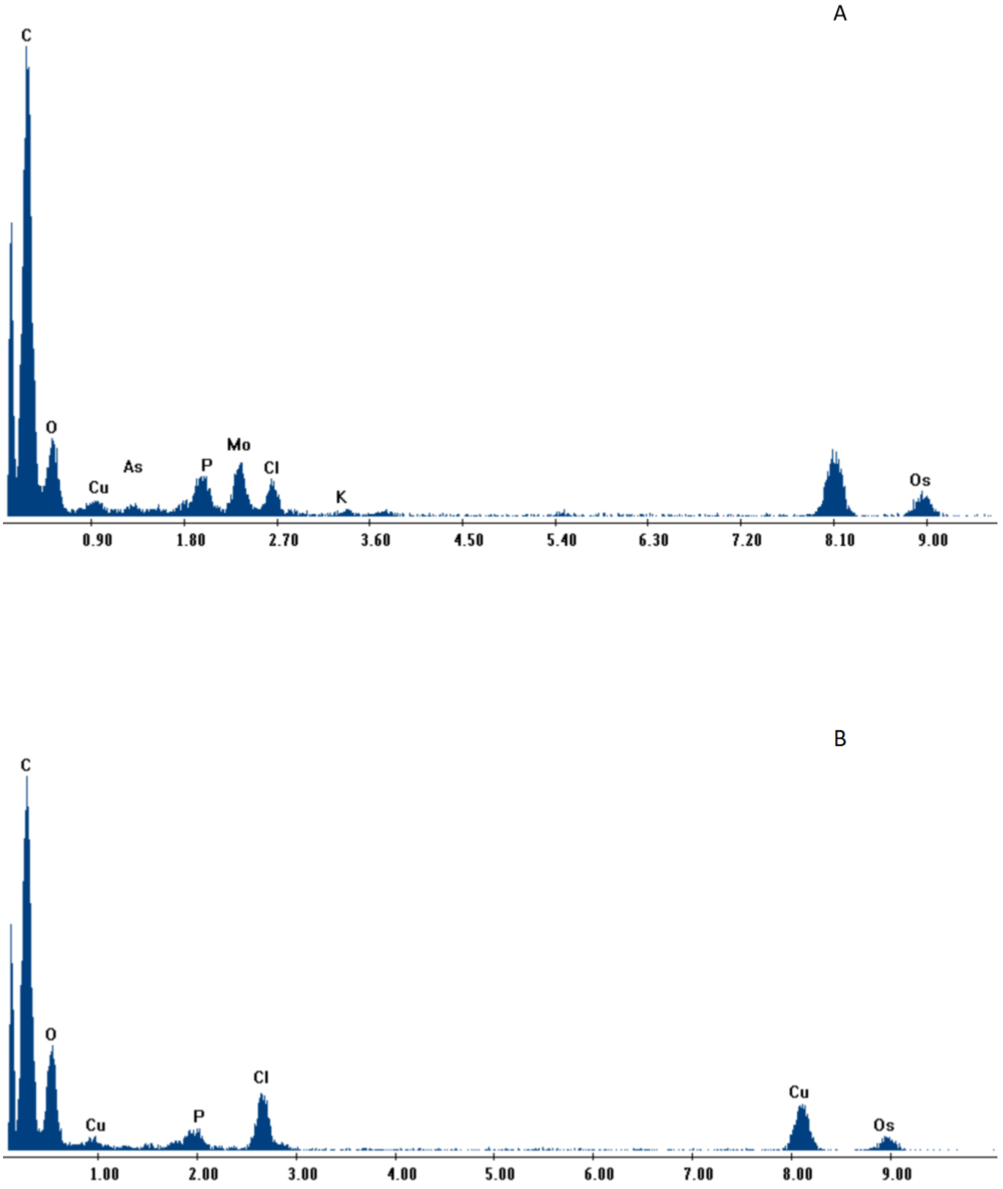

(Fig. S4)

Fig. S5. The polar lipid profiles stained with molybdophosphoric acid after separation by two-dimensional thin-layer chromatography.

Strains M0-14^{T} (a), *C. cellasea* KACC 20548^{T} (b), *A. fermentans* KACC 20763^{T} (c), *C. bogoriensis* KACC 20567^{T} (d), *P. sicca* LRZ-2^{T} (from Kim *et al.* [2]) (e); DPG, diphosphatidylglycerol; PC, phosphatidylcholine; PG, phosphatidylglycerol; PIM, phosphatidylinositol mannosides; PE, phosphatidylethanolamine; PME, phosphatidylmethyl ethanolamine; PI, phosphatidylinositol; PIDM, phosphatidylinositol dimannoside; PGL, unknown phosphoglycolipids; PL, unknown phospholipids; APL, unknown aminophospholipids; AL, unknown aminolipid; L, unknown lipid.

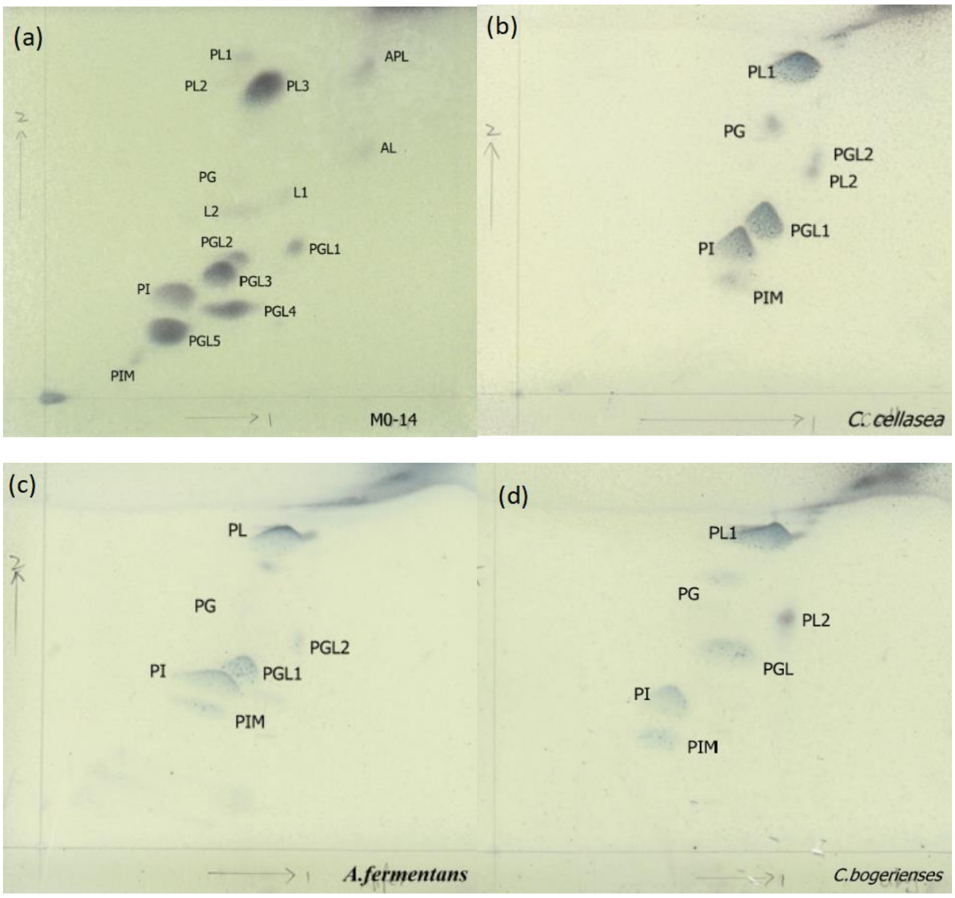

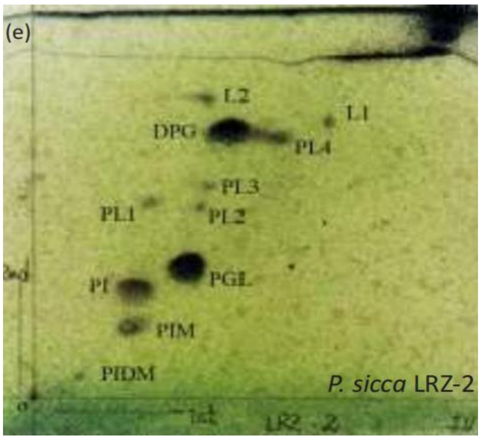

(Fig. S5)

Table S1. Cellular fatty acid compositions of strain M0-14^{T} and type strains of members of the genera *Cellulomonas* and *Actinotalea*. tr, trace (<0.5 %); ND, not detected.

Fatty acid (%)	M0-14^{T}	*P. sicca* LRZ-2^{T}	*C. cellasea* KACC 20548^{T}	*A. fermentans* KACC 20763^{T}	*C. chitinilytica* DSM 17922^{T}	*C. bogoriensis* KACC 20567^{T}
anteiso-$C_{13:0}$	1.0	Tr	3.4	1.9	tr	0.8
iso-$C_{14:0}$	2.4	2.0	12.2	9.3	6.7	0.8
$C_{14:0}$	3.4	tr	7.5	11.9	1.1	6.5
anteiso-$C_{15:1}$ A	2.9	13.1	2.7	1.1	7.2	5.7
iso-$C_{15:0}$	0.6	5.2	7.1	8.2	7.8	1.4
anteiso-$C_{15:0}$	36.9	39.5	48.8	41.5	50.2	48.4
iso-$C_{16:0}$	2.5	23.9	6.1	5.9	3.9	3.5
$C_{16:0}$	20.2	1.9	9.1	14.8	7.2	24.8
$C_{16:1}\ \omega 9c$	2.6	ND	ND	ND	ND	ND
iso-$C_{17:0}$	ND	1.3	tr	tr	5.8	tr
anteiso-$C_{17:0}$	4.6	11.0	1.2	tr	4.3	4.6
$C_{17:0}$	1.2	ND	ND	tr	1.9	0.7
$C_{17:1}\ \omega 8c$	1.3	ND	ND	ND	ND	ND
$C_{17:1}\ \omega 6c$	1.1	ND	ND	ND	ND	ND
$C_{18:0}$	1.3	tr	tr	tr	1.7	0.8
$C_{18:1}\ \omega 9c$	2.0	tr	tr	0.7	tr	0.5
Summed feature 3	10.9	ND	tr	1.3	ND	0.8
Summed feature 8	1.9	ND	tr	tr	tr	ND

Summed features are fatty acids that cannot be resolved reliably from another fatty acid using the chromatographic conditions chosen. The MIDI system groups these fatty acids together as one feature with a single percentage of the total. Summed feature 3 contains $C_{16:1}\ \omega 7c$ and/or $C_{16:1}\ \omega 6c$; summed feature 8 contains $C_{18:1}\ \omega 7c$ and/or $C_{18:1}\ \omega 6c$ [25].

Table S2. The digital DNA-DNA hybridization (dDDH) and average nucleotide identity (ANI) values between strain M0-14^{T} and closely related strains.

*Results are percentages based on calculations using Formula 2: The sum of all identities found in high-scoring segment pairs (HSPs) were divided by the overall HSP length. Formula 2 which is independent of genome length and thus is more robust when applied to incomplete draft genomes.

Strains	dDDH (%)*	ANI (%)	G+C difference
Pengzhenrongella sicca LRZ-2^{T}	23.1	79.97	1.57
Actinotalea fermentans NBRC 105374	19.8	74.22	3.26
Cellulomonas aerilata NBRC 106308	21.2	76.95	3.56
Cellulomonas bogoriensis DSM 16987	19.3	73.84	1.39
Cellulomonas cellasea NBRC 3753	21.1	76.8	4.01
Cellulomonas chitinilytica NBRC 110799	19.8	75.37	2.62
Cellulomonas denverensis ATCC BAA-788	19.6	74.5	2.08
Cellulomonas taurus P40-2	19.6	74.82	1.15
Cellulomonas timonensis SN7	20	75.64	1.57
Luteimicrobium subarcticum DSM 22413	19.6	73.89	2.34
